# Genetic variations and polymorphisms in the ezrin gene are associated with age-related cataract

**Published:** 2013-07-20

**Authors:** Qinghong Lin, Nan Zhou, Na Zhang, Bidan Zhu, Shanshan Hu, Zhou Zhou, Yanhua Qi

**Affiliations:** Department of Ophthalmology, the Second Affiliated Hospital of Harbin Medical University, Harbin, China

## Abstract

**Purpose:**

Age-related cataract (ARC) is a complex multifactorial disorder, including genetic and environmental factors. Ezrin (*EZR*), a member of the ezrin/radixin/moesin (ERM) protein family, plays a crucial role in the development of the lens as a plasma membrane–cytoskeleton linker. We conducted this study to investigate the role of genetic variations of ezrin and the relationship between single nucleotide polymorphisms (SNPs) in *EZR* and susceptibility to ARC in a Chinese population.

**Methods:**

A total of 205 sporadic age-related cataract patients and 218 unrelated random healthy controls participated in our study. Genomic DNA was extracted from peripheral blood leukocytes. All exons of EZR were sequenced after being amplified with polymerase chain reaction. The functional consequences of the mutations were analyzed using PolyPhen2. SNP statistical analysis was performed using SNPstats.

**Results:**

We found three novel variations in 205 patients. None presented in the 218 controls, including c.441C>G, c.924G>C, and c.1503G>A. PolyPhen2 predicted that the c.924G>C mutation probably had pathogenicity. Compared with the healthy controls, the rs5881286 -/GT genotype and - allele frequencies (p=0.0012; odds ratio [OR]=3.37; 95% confidence interval [CI]=1.70–6.70; p=3.96e-5; χ^2^=18.98, respectively), rs2242318 T/C genotype and C allele frequencies (p=0.0045; OR=3.40; 95% CI=1.70–6.79; p=8.82e-6; χ^2^=21.86, respectively), and rs144581330 A/G genotype and G allele frequencies (p=0.0472; OR=14.46; 95% CI=1.29–162.43; p=0.0244, χ^2^=6.99, respectively) were higher in the patients with age-related cataract. SNP rs144581330 in exon 2 was also predicted to be probably damaging by PolyPhen2. Haplotype association including the - allele of rs5881286, C allele of rs2242318, and A allele of rs144581330 exhibited significantly higher distribution in the patients with ARC (p=8.0e-4; OR=3.38; 95% CI=1.66–6.87).

**Conclusions:**

This study suggests that the genetic variations and SNPs in the gene *EZR* possibly contribute to the development of age-related cataracts in the Chinese population.

## Introduction

Cataract is one of the most common causes of vision impairment and blindness all over the world. As the world’s population ages, visual dysfunction and blindness induced by age-related cataract (ARC) are increasing. Since ARC is a multifactorial disease, environmental components and genetic predisposition contribute to the development of the pathological condition. Increased age and female gender are associated with an increased risk for all types of ARC. Current cigarette smoking is the most significant risk factor for nuclear cataracts; higher systolic blood pressure, a history of cigarette smoking, and a history of diabetes for cortical cataracts; higher systolic blood pressure for posterior subcapsular cataracts [1]. In addition, ultraviolet radiation exposure, sunlight exposure, drug ingestion, and lower body mass index are also risk factors for ARC [2,3]. In the past few years, research into ARC has focused on the genetic factor and established that mutations that severely disrupt the lens cell architecture or environment might result in congenital cataracts, while relatively mild mutations might produce age-related cataract [4]. Strong evidence from twin studies has demonstrated the contribution of genetic factors in the pathogenesis of age-related cataract, a heritability of 53%–58% for cortical cataract and 48% for nuclear cataract [5,6]. To date, many genetic studies have revealed variations or polymorphisms in genes may be associated with ARC [7-13].

Crystalline lens transparency is achieved and maintained by precise organized cell membrane domains and the cell–cell interactions. The ability to organize specialized membrane domains is essential to most cells, including the epithelial cells and the fiber cells of the lens. Mutations that severely disrupt it might produce congenital cataract and age-related cataract. Ezrin (encoded by the ezrin [*EZR*] gene) is a member of the ezrin/radixin/moesin (ERM) protein family. This protein serves as an intermediate between the plasma membrane and the actin cytoskeleton and has been implicated in various human cancers [14,15]. Ezrin has a homologous amino acid domain called the FERM (4.1 protein/ezrin/radixin/moesin) domain, an α-helical region, and a carboxy-terminal ERM-associated domain (C-ERMAD) tail. This domain is involved in many important roles: maintenance of cell shape, cell mobility, and membrane trafficking [16]. In the lens, ezrin exists as part of the ezrin, periaxin, periplakin, and desmoyokin (EPPD) complex and is important in cell adhesive interactions and cell–cell junction of fiber cells. The EPPD complex plays an important role in the maintenance of lens structure and functions. The dysfunction of ezrin may cause aberrant lens development [17,18]. Ezrin has specific cross-linking with Aquaporin 0 (AQP0). The interaction is between the C-terminus of AQP0 and the subdomains of ezrin. It is expected to occur only in new differentiating fiber cells because ezrin is degraded in the nucleus. This suggests that AQP0 may be a candidate ezrin-binding part in the EPPD complex and presumes the way that the EPPD complex links to the plasma membrane. Thus, reduction in or dysfunction of ezrin would potentially cause disorder of the fiber cells that induce opacification of the lens [19,20]. Given this background, we chose *EZR* as a potential ARC candidate gene in this study.

To date, neither mutations in ezrin nor single nucleotide polymorphisms (SNPs) in *EZR* have been reported to be associated with cataract patients. In this case-control study, we screened all exons and the flanking region of *EZR* to investigate the genetic variations of ezrin and the relationship between SNPs in *EZR* and susceptibility to ARC in Chinese population.

## Methods

### Participants and ethics statement

Two hundred five patients with age-related cataracts and 218 unrelated healthy controls were recruited from the Second Affiliated Hospital Ophthalmic Clinic of Harbin Medical University (Table 1). Patients and controls were matched on gender (χ^2^ test, p=0.7027). Differences in age were adjusted with logistic regression.

**Table 1:** Baseline Characteristics of the Study Subjects.

**Group**	**Total**	**Male (%)**	**Female (%)**	**Mean Age(yo)**
Patients	205	94 (45.85)	111 (54.15)	71.59+8.18
Controls	218	104 (47.71)	114 (52.29)	53.29±8.41
		χ^2^ test, p=0.7027		*t* test, p=6.62e-75

Cataract diagnosis was determined according to the Lens Opacities Classification System III (LOCSIII) [21]. All patients had bilateral cataracts, and the severity of the cortical or nuclear cataracts was greater than grade II. Patients with secondary cataracts due to trauma, toxins, inflammation, and degenerative ocular diseases were excluded from the study. In addition, patients who smoked, used alcohol, had exposure to ultraviolet B radiation, or took medication such as steroids and those with diabetes, hypertension, glaucoma, high myopia, and any other syndrome were not considered. Unrelated healthy individuals from the same ethnic background without a history of cataracts served as normal controls. The control subjects were selected randomly during a routine medical fitness examination, which included an ophthalmic examination. The bilateral lenses of the controls were all classified with LOCS grade 0.1. Written informed consent was obtained from all subjects after the nature and possible consequences of the study were explained. All experiments were approved by the Institutional Review Board of Harbin Medical University (Harbin, China) and conducted according to the principles in the Declaration of Helsinki.

### Deoxyribonucleic acid isolation and genotyping

Five milliliter samples of venous blood were collected in EDTA vacutainers (BD, San Jose, CA) from the patients with ARC and the control subjects. Genome DNA was extracted from peripheral blood leukocytes using the QIAamp DNA Blood Mini Kits (Qiagen Science, Germantown, MD). The primers (Table 2) for polymerase chain reaction (PCR) were designed using Primer3 according to the reference sequences in the NCBI Gene database. The PCR conditions were 95 °C for 4 min, followed by 35 cycles of 94 °C for 30 s, 55 °C for 30 s, 72 °C for 30 s, and a final extension step at 72 °C for 7 min. We sequenced the PCR products with the ABI3730 Automated Sequencer (PE Biosystems, Foster City, CA) and analyzed the sequencing results with Lasergene SeqMan (DNASTAR, Madison, WI).

**Table 2:** Primers Used for Polymerase Chain Reaction (PCR) in This Study

**Exon**	**Forward(5′-3′)**	**Rverse(5′-3′)**	**Amplicon Location(hg19)**
Exon −1	TTCTGCTCTGACTCCAGGTTG	TGTGGTCGGAAGCCATCT	chr6:159238738–159239215
Exon −2	GCCTCCTGAGACATCCC	TGAATCCGCCTGACTTT	chr6:159210069–159210587
Exon −3	CTCCCATGGTTCTGTGTGTG	GAAGCAGACCAACACCCAAT	chr6:159208088–159208583
Exon −4	GCCCCATTGAGTCTTGGTTA	GCATGAAGGAGCACTTGACA	chr6:159206216–159206706
Exon −5	TGGCATAGCAAACTTCTC	TTAACTCGGTTCCCTCA	chr6:159205544–159206117
Exon −6	GACTCTGCCTGTTTCACTC	ATTCTGCCTTCCTCTACC	chr6:159204304–159204728
Exon −7	GGGAATGAACAGCAGAA	GACTATCACTGGCTACTCG	chr6:159197340–159197881
Exon −8	ATTCCTAGTGCCGAGATGC	CTCCAGGGCTGACAACA	chr6:159192083–159192580
Exon −9	ACCACGCTGGGATCTTC	TTCGGCTGTGAGTCTGC	chr6:159191665–159192155
Exon −10	CGTTGGACGGGTTTCTAG	GGCAATCTTGGCAGTGTAT	chr6:159190415–159191189
Exon −11	GCGGTGGATCAGATAAAGA	GCTGGTATTGGCAGAGGA	chr6:159190192–159190848
Exon −12	CAGGACAGAGGGAGGTGA	ACATTAAGCAGCATTGGTCTA	chr6:159188116–159188633
Exon −13	TCTAGTGAGGGCATCCG	TTCCGTAATTCAATCAGTCC	chr6:159187671–159188379

### PolyPhen2 analysis

PolyPhen2 online software is a revised version of PolyPhen for annotating coding nonsynonymous SNPs on the function of encoded proteins by performing various sequence and structure analyses, yielding a probability of altered function. The software is based on the position-specific independent counts (PSIC) score derived from multiple sequence alignments of observations [22]. We used the data set HumDiv to test and train the predictions made by PolyPhen2. Predictions were characterized as either benign, possibly damaging, or probably damaging on the output. We downloaded the amino acid sequences from the NCBI HomoloGene database and evaluated the cross-species conservation of the nonsynonymous mutations with Lasergene MegAlign (DNASTAR).

### Statistical analysis

All the SNPs were validated with the NCBI SNP database and 1000 Genomes. The allele and genotype frequencies of the *EZR* SNPs were compared between the patients and controls. We evaluated the frequency of the genotypes and alleles in this study using the χ^2^ test and logistic regression. Paired SNP linkage disequilibrium analysis and haplotype analysis were performed as well. The risk for patients was estimated using the odds ratio (OR) and 95% confidence interval (CI). Statistical analysis was performed using SNPstats [23]. A p<0.05 was considered statistically significant.

## Results

### Characteristics of participants

Two hundred five patients with age-related cataracts and 218 unrelated healthy controls were recruited in this study. The mean age of the cases was 71.59+8.18 years and that of controls was 53.29+8.41 years. In addition, the patients and controls were matched on gender (χ^2^ test, p=0.7027, Table 1). Differences in age were adjusted with logistic regression.

### Identification of novel mutations and single nucleotide polymorphisms

**Figure 1:**
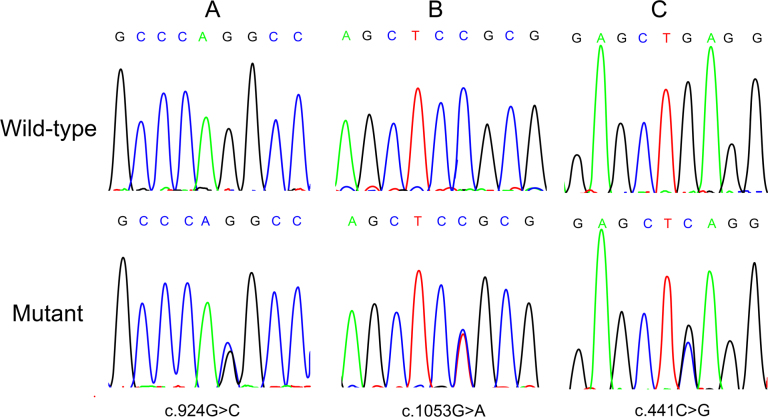
Mutation analysis of the *EZR* gene. The forward strand sequence chromatogram (**A**) shows the variation c.924G>C we found in exon 8. The reverse strand sequence chromatograms (**B**, **C**) show the mutations c.1503G>A in exon 12 and c.441C>G in exon 4.

Three novel nucleotide changes, c.441C>G, c.924G>C, and c.1503G>A in exon 4, exon 8, and exon 12, respectively, were found in the patients, and none presented in the normal controls (Figure 1, Table 3). Among these changes, the nonsynonymous change c.924G>C may lead to p.Q308H. Compared with the healthy controls, the rs5881286 -/GT genotype and - allele, rs2242318 T/C genotype and C allele, and rs144581330 A/G genotype and G allele were observed with significantly higher frequencies in the patients with age-related cataract. The GT/GT genotype of rs5881286, the T/T genotype of rs2242318, and the A/A genotype of rs144581330 were in all three patients in whom we found novel nucleotide changes. Table 4 shows the distribution of the genotype and allele frequencies of rs5881286, rs2242318, and rs144581330 in this study. The distributions of all SNPs were consistent with the Hardy–Weinberg equilibrium (HWE) in the control group. After the laboratory work, we reexamined the patients carrying mutations and confirmed the patients had no family history of cataracts.

**Table 3:** Clinical Data of Patients Who Carried Genetic Variation

**Serial number**	**Gender**	**Age(yo)**	**Type(both eyes)**	**Variation**	**Genotype**
A	Female	72	Cortical	c.924G>C	heterozygous
B	Female	57	Mix	c.1503G>A	heterozygous
C	Male	59	Nuclear	c.441C>G	heterozygous

**Table 4:** Distribution of the Genotype and Allele Frequencies of rs5881286, rs2242318 and rs144581330 in the Patient and Control Group (adjusted by age).

**SNP**	**Patient/control**	**Genotype N (%)**			**Allele N (%)**	
rs5881286		GT/GT	-/GT	−/−	GT	-
	Patients	127 (62%)	78 (38%)	0 (0%)	332 (81%)	78 (19%)
	Controls	180 (83%)	38 (17%)	0 (0%)	398 (91%)	38 (9%)
		p=0.0012 OR*(95% CI^#^)=3.37 (1.70–6.70)			χ^2^=18.98,p=3.96e-5	
rs2242318		T/T	T/C	C/C	T	C
	Patients	126 (61.5%)	75 (36.6%)	4 (2%)	327 (80%)	83 (20%)
	Controls	180 (82.6%)	37 (17%)	1 (0.5%)	397 (91%)	39 (9%)
		p=0.0045 OR(95% CI)=3.40 (1.70–6.79)			χ^2^=21.86, p=8.82e-6	
rs144581330		A/A	A/G	G/G	A	G
	Patients	196 (95.6%)	9 (4.4%)	0 (0%)	401 (98%)	9 (2%)
	Controls	217 (99.5)	1 (0.5%)	0 (0%)	435 (100%)	1 (0%)
		p=0.0472 OR(95% CI)=14.46 (1.29–162.43)			χ^2^=6.99, p=0.0244	

* Odds Ratio # Confidence Interval p values were adjusted by Bonferroni Correction

### Effects of nonsynonymous changes and cross-species conservation with in silico analysis

**Figure 2:**
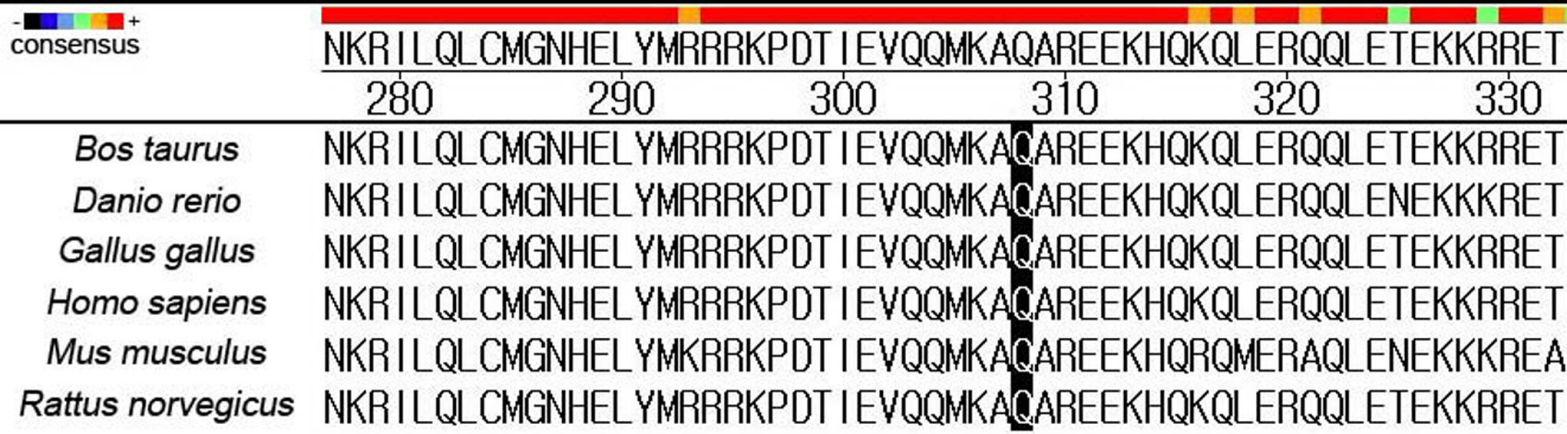
Cross-species conservation analysis. The black bars highlight the interesting positions of the proteins. Multiple alignments indicate that glutamine at codon 308 in ezrin is highly conserved.

By using PolyPhen2, p.Q308H in *EZR* was predicted to be probably damaging. SNP rs144581330 in exon 2, which causes the p.N6S substitution, was also predicted to be probably damaging by PolyPhen2. The scores and results of PolyPhen2 are listed in Table 5. We aligned the amino acid sequences of the *EZR* gene from several species. The glutamine at codon 308 in *EZR* was highly conserved (Figure 2).

**Table 5:** Variations Found in *EZR* and PolyPhen2 Results of the Nonsynonymous Mutations

**Nucleotide notation**	**Protein notation**	**Polyphen2 score**	**Polyphen2 prediction**
c.441C>G	synonymous	-	-
c.924G>C	p.Q308H	1.000	Probably damaging
c.1503G>A	synonymous	-	-
c.17A>G	p.N6S	0.992	Probably damaging

### Paired single nucleotide polymorphism linkage disequilibrium analysis and haplotype analysis

**Figure 3:**
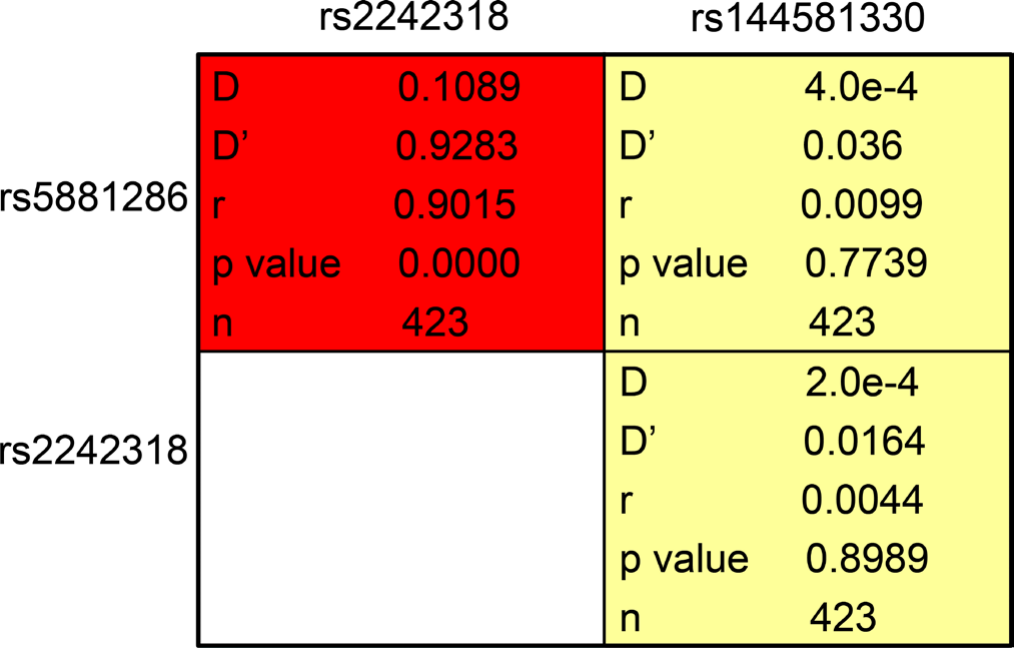
. Paired single nucleotide polymorphism linkage disequilibrium analysis. Paired SNP linkage disequilibrium analysis revealed that rs5881286 was in linkage disequilibrium with rs2242318 (D’>0.75).

Paired SNP linkage disequilibrium analysis revealed that rs5881286 was in linkage disequilibrium with rs2242318 (D’>0.75; Figure 3). As shown in Table 6, haplotype association analysis denoted that the haplotype association including the - allele of rs5881286, the C allele of rs2242318, and the A allele of rs144581330 exhibited significantly higher distribution in the patients compared to the normal controls (p=8.0e-4; OR=3.38; 95% CI=1.66–6.87).

**Table 6:** Haplotype Association Analysis (adjusted by age).

**rs5881286**	**rs2242318**	**rs144581330**	**Patient Frequency**	**Control Frequency**	**Odds Ratio**	**P Value**
					**(95% Confidence Interval)**	
GT	T	A	0,766	0,8376	1	—
-	C	A	0,1747	0,0825	3.38 (1.66–6.87)	8,00E-04
GT	C	A	0,0248	0,0069	2.77 (0.37–20.53)	0,32

## Discussion

Lens epithelial cell and fiber cell elongation and differentiation are associated with dramatic changes in cell morphology, membrane architecture, cortical cytoskeletal organization, and cell–cell adhesions. The ERM protein family is part of the band 4.1 superfamily that maintains the cell membrane domains. These proteins are expressed in a developmental and tissue-specific manner, with most epithelial cells predominantly expressing ezrin and endothelial cells predominantly expressing moesin. The proteins are involved in many important roles and can regulate the activities of the signal transduction pathways [24,25].

The *EZR* gene consists of 13 exons. It encodes ezrin with 586 amino acids. Ezrin, similar to other ERM protein members, has a homologous amino acid domain called the FERM domain and terminates in the C-ERMAD as a tail [26]. The N-terminal FERM domain consists of three subdomains, named F1, F2, and F3 [27]. This domain followed by an approximate 150 residue region predicted to have a high α-helical content containing three helices (αA, αB, and αC) and characterized by assembly into a coiled-coil structure. Ezrin has a proline residue rich linker region and C-ERMAD, which contains the F-actin-binding site. As the plasma membrane–cytoskeleton linker, C-ERMAD binds with membrane proteins such as CD44 through an N-terminal FERM domain and connects to F-actin through a C-terminal domain.

The ezrin protein exists in a dormant, closed conformation as the binding of the FERM domain to the C-ERMAD. Therefore, the ligand binding sites, including F-actin and the scaffolding protein EBP50, are masked [28,29]. Ezrin is activated through PtdIns (4, 5) P_2_ binding and phosphorylation of threonine 567, which reduces the affinity of the N-terminal FERM domain for C-ERMAD. The activation makes these functional domains ready for binding proteins. Studies focused on the structure of the ERM protein family proposed the α-helical domain of the family bends at the αA-αB junction and again at the αB-αC junction in a closed conformation. That causes the C-ERMAD to be positioned over F2 and F3 of the FERM domain [30,31]. This indicates that the closed conformation and its activation are largely mediated by the central α-helical domain. Thus, the α-helical region is significant to the stability and activation of dormant ezrin monomers.

**Figure 4:**
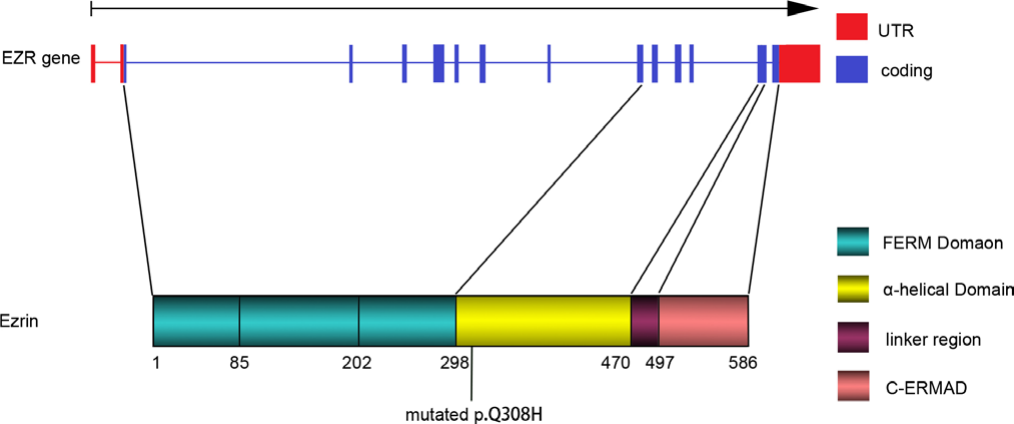
Genomic structure of the EZR exons, domain organization of ezrin and the identified mutation. The red parts indicate the UTR of the *EZR* gene, and the blue domains indicate the exons that are translated (upper panel). Ezrin consists of the FERM domain (composed of three subdomains, designated F1, F2, and F3), α-helical domain, linker region, and C-ERMAD (lower panel). The c.924G>C in EZR exon 8 and the p.Q308H substitution in ezrin are traced at the bottom of the figure.

Another noticeable feature of ERM proteins is their ability to form homodimers or heterodimers [32]. It suggests dimers may consist of antiparallel subunits held together by two FERM/C-ERMAD associations [33]. This indicates that a parallel association in which the central α-helical region assembles a coiled-coil drives dimer formation yielding a molecule with two FERM domains on one end and two C-ERMADs on the other, linking from the membrane proteins to the cytoskeleton [34]. The coiled-coil formed by the α-helical region and the FERM/C-ERMAD associations are essential for dimer formation [35]. In addition, this region could serve as a binding site for associated molecules, including the regulatory subunit of protein kinase A, which provides specific regulation of plasma membrane pumps and channels located in the proximity of subplasmalemmal F-actin [36]. That means the mutation in this α-helical region could impact the ability of ezrin to be the nidus for the localization of critical regulatory enzyme systems, apart from linking the plasma membrane with the cytoskeleton. In our study, the mutation c. 924G>C that resulted in a p.Q308H substitution is in the α-helical domain (Figure 4). The amino acid affected by this mutation in the α-helical region evolutionarily is conserved at a high level. This mutation may impact the stability of the coiled-coil structure, affecting the release of the function region and the conformation of the ezrin monomer or dimer. However, the specific mechanism the mutation influences on ezrin is still unknown. Synonymous variations may still contribute to the development of human diseases [37]. Therefore, the synonymous mutations c.441C>G and c.1503G>A found in *EZR* may affect the normal process of translation.

In this study, we also investigated the association between gene *EZR* polymorphisms and the risk of age-related cataract in the Chinese population. We found a statistically significant difference in the *EZR* polymorphism frequencies between the patients and normal controls. The data show the polymorphisms of the -/GT genotype and the - allele of rs5881286, the T/C genotype and the C allele of rs2242318, and the A/G genotype and the G allele of rs144581330 may be associated with an increased risk of cataract. In addition, the paired SNP linkage disequilibrium analysis revealed that rs5881286 was in linkage disequilibrium with rs2242318 and the haplotype association including the - allele of rs5881286, the C allele of rs2242318, and the A allele of rs144581330 exhibited significantly higher distribution in patients than in controls. That meant the GT allele of rs5881286, the T allele of rs2242318, and the A allele of rs144581330 may have a protective effect against the development of age-related cataract, particularly when the GT allele of rs5881286 and the T allele of rs2242318 are in one person.

In the past few years, several studies showed that variations in the untranslated region (UTR) were important to translational regulation [38]. The rs5881286 in the 3′ UTR of *EZR* potentially interferes with the process of candidate microRNA binding to the *EZR* gene, including hsa-miR-548u, hsa-miR-4744, hsa-miR-4801, and hsa-miR-4731–3p. This may impact the normal process of translation. This polymorphism could also lead to significant alteration of the secondary mRNA structure, forming a new loop-stem structure and affecting the stability of the secondary mRNA structure. The rs144581330 located in the coding region causes the p.N6S. It was predicted to be probably damaging by PolyPhen2. In addition, this amino substitution may affect the normal function of ezrin since its location is in the FERM domain. However, further studies are needed to find the precise mechanisms.

This is the first study of *EZR* and age-related cataract development in the Chinese population. No mutation in ezrin has been reported in age-related cataract or congenital cataract as a pathogenic gene before. The current knowledge of the highly conserved α-helical region in ezrin is rudimentary. Further study for a better understanding of this protein is still needed.

## References

[1] Tsai SY, Hsu WM, Cheng CY, Liu JH, Chou P (2003). Epidemiologic study of age-related cataracts among an elderly Chinese population in Shih-Pai, Taiwan.. Ophthalmology.

[2] Nirmalan PK, Krishnadas R, Ramakrishnan R, Thulasiraj RD, Katz J, Tielsch JM, Robin AL (2003). Lens opacities in a rural population of southern India: the Aravind Comprehensive Eye Study.. Invest Ophthalmol Vis Sci.

[3] Krishnaiah S, Vilas K, Shamanna BR, Rao GN, Thomas R, Balasubramanian D (2005). Smoking and its association with cataract: results of the Andhra Pradesh eye disease study from India.. Invest Ophthalmol Vis Sci.

[4] Hejtmancik JF, Kantorow M (2004). Molecular genetics of age-related cataract.. Exp Eye Res.

[5] Hammond CJ, Duncan DD, Snieder H, de Lange M, West SK, Spector TD, Gilbert CE (2001). The heritability of age-related cortical cataract: the twin eye study.. Invest Ophthalmol Vis Sci.

[6] Hammond CJ, Snieder H, Spector TD, Gilbert CE (2000). Genetic and environmental factors in age-related nuclear cataracts in monozygotic and dizygotic twins.. N Engl J Med.

[7] Jun G, Guo H, Klein BE, Klein R, Wang JJ, Mitchell P, Miao H, Lee KE, Joshi T, Buck M, Chugha P, Bardenstein D, Klein AP, Bailey-Wilson JE, Gong X, Spector TD, Andrew T, Hammond CJ, Elston RC, Iyengar SK, Wang B (2009). EPHA2 is associated with age-related cortical cataract in mice and humans.. PLoS Genet.

[8] Liu Y, Ke M, Yan M, Guo S, Mothobi ME, Chen Q, Zheng F (2011). Association between gap junction protein-alpha 8 polymorphisms and age-related cataract.. Mol Biol Rep.

[9] Zuercher J, Neidhardt J, Magyar I, Labs S, Moore AT, Tanner FC, Waseem N, Schorderet DF, Munier FL, Bhattacharya S, Berger W, Kloeckener-Gruissem B (2010). Alterations of the 5′untranslated region of SLC16A12 lead to age-related cataract.. Invest Ophthalmol Vis Sci.

[10] Bhagyalaxmi SG, Srinivas P, Barton KA, Kumar KR, Vidyavathi M, Petrash JM, Bhanuprakash Reddy G, Padma T (2009). A novel mutation (F71L) in alphaA-crystallin with defective chaperone-like function associated with age-related cataract.. Biochim Biophys Acta.

[11] Okano Y, Asada M, Fujimoto A, Ohtake A, Murayama K, Hsiao KJ, Choeh K, Yang Y, Cao Q, Reichardt JK, Niihira S, Imamura T, Yamano T (2001). A genetic factor for age-related cataract: identification and characterization of a novel galactokinase variant, “Osaka,” in Asians.. Am J Hum Genet.

[12] Zhou J, Hu J, Guan H (2010). The association between copy number variations in glutathione S-transferase M1 and T1 and age-related cataract in a Han Chinese population.. Invest Ophthalmol Vis Sci.

[13] Zhou Z, Wang B, Hu S, Zhang C, Ma X, Qi Y (2011). Genetic variations in GJA3, GJA8, LIM2, and age-related cataract in the Chinese population: a mutation screening study.. Mol Vis.

[14] Li Q, Gao H, Xu H, Wang X, Pan Y, Hao F, Qiu X, Stoecker M, Wang E, Wang E (2012). Expression of ezrin correlates with malignant phenotype of lung cancer, and in vitro knockdown of ezrin reverses the aggressive biological behavior of lung cancer cells.. Tumour Biol.

[15] Saito S, Yamamoto H, Mukaisho K, Sato S, Higo T, Hattori T, Yamamoto G, Sugihara H (2013). Mechanisms underlying cancer progression caused by ezrin overexpression in tongue squamous cell carcinoma.. PLoS ONE.

[16] Louvet-Vallée S (2000). ERM proteins: from cellular architecture to cell signaling.. Biol Cell.

[17] Straub BK, Boda J, Kuhn C, Schnoelzer M, Korf U, Kempf T, Spring H, Hatzfeld M, Franke WW (2003). A novel cell-cell junction system: the cortex adhaerens mosaic of lens fiber cells.. J Cell Sci.

[18] Maddala R, Skiba NP, Lalane R, Sherman DL, Brophy PJ, Rao PV (2011). Periaxin is required for hexagonal geometry and membrane organization of mature lens fibers.. Dev Biol.

[19] Bagchi M, Katar M, Lo WK, Yost R, Hill C, Maisel H (2004). ERM proteins of the lens.. J Cell Biochem.

[20] Wang Z, Schey KL (2011). Aquaporin-0 interacts with the FERM domain of ezrin/radixin/moesin proteins in the ocular lens.. Invest Ophthalmol Vis Sci.

[21] Chylack LT, Wolfe JK, Singer DM, Leske MC, Bullimore MA, Bailey IL, Friend J, McCarthy D, Wu SY (1993). The Lens Opacities Classification System III.. Arch Ophthalmol.

[22] Sunyaev S, Ramensky V, Koch I, Lathe W, Kondrashov AS, Bork P (2001). Prediction of deleterious human alleles.. Hum Mol Genet.

[23] Solé X, Guino E, Valls J, Iniesta R, Moreno V (2006). SNPStats: a web tool for the analysis of association studies.. Bioinformatics.

[24] Bretscher A, Chambers D, Nguyen R, Reczek D (2000). ERM-Merlin and EBP50 protein families in plasma membrane organization and function.. Annu Rev Cell Dev Biol.

[25] Bretscher A, Edwards K, Fehon RG (2002). ERM proteins and merlin: integrators at the cell cortex.. Nat Rev Mol Cell Biol.

[26] Chishti AH, Kim AC, Marfatia SM, Lutchman M, Hanspal M, Jindal H, Liu SC, Low PS, Rouleau GA, Mohandas N, Chasis JA, Conboy JG, Gascard P, Takakuwa Y, Huang SC, Benz EJ, Bretscher A, Fehon RG, Gusella JF, Ramesh V, Solomon F, Marchesi VT, Tsukita S, Tsukita S, Hoover KB (1998). The FERM domain: a unique module involved in the linkage of cytoplasmic proteins to the membrane.. Trends Biochem Sci.

[27] Pearson MA, Reczek D, Bretscher A, Karplus PA (2000). Structure of the ERM protein moesin reveals the FERM domain fold masked by an extended actin binding tail domain.. Cell.

[28] Gary R, Bretscher A (1995). Ezrin self-association involves binding of an N-terminal domain to a normally masked C-terminal domain that includes the F-actin binding site.. Mol Biol Cell.

[29] Reczek D, Bretscher A (1998). The carboxyl-terminal region of EBP50 binds to a site in the N-terminal domain of ezrin that is masked in the dormant molecule.. J Biol Chem.

[30] Li Q, Nance MR, Kulikauskas R, Nyberg K, Fehon R, Karplus PA, Bretscher A, Tesmer JJ (2007). Self-masking in an intact ERM-merlin protein: an active role for the central alphα-helical domain.. J Mol Biol.

[31] Hennigan RF, Foster LA, Chaiken MF, Mani T, Gomes MM, Herr AB, Ip W (2010). Fluorescence resonance energy transfer analysis of merlin conformational changes.. Mol Cell Biol.

[32] Gary R, Bretscher A (1993). Heterotypic and homotypic associations between ezrin and moesin, two putative membrane-cytoskeletal linking proteins.. Proc Natl Acad Sci USA.

[33] Bretscher A, Gary R, Berryman M (1995). Soluble ezrin purified from placenta exists as stable monomers and elongated dimers with masked C-terminal ezrin-radixin-moesin association domains.. Biochemistry.

[34] Turunen O, Sainio M, Jaaskelainen J, Carpen O, Vaheri A (1998). Structure-function relationships in the ezrin family and the effect of tumor-associated point mutations in neurofibromatosis 2 protein.. Biochim Biophys Acta.

[35] Chambers DN, Bretscher A (2005). Ezrin mutants affecting dimerization and activation.. Biochemistry.

[36] Dransfield DT, Bradford AJ, Smith J, Martin M, Roy C, Mangeat PH, Goldenring JR (1997). Ezrin is a cyclic AMP-dependent protein kinase anchoring protein.. EMBO J.

[37] Kimchi-Sarfaty C, Oh JM, Kim IW, Sauna ZE, Calcagno AM, Ambudkar SV, Gottesman MMA (2007). “silent” polymorphism in the MDR1 gene changes substrate specificity.. Science.

[38] Chatterjee S, Pal JK (2009). Role of 5′- and 3′-untranslated regions of mRNAs in human diseases.. Biol Cell.

